# Supplementary material for: Brochoscopic Airway Clearance Therapy vs. Conventional Sputum Aspiration: The Future of Flexible Brochoscopes in Intensive Care Units?
Source: Diagnostics (Basel). 2023 Oct 22;13(20):3276. doi: 10.3390/diagnostics13203276 (PMC10606468; doi:10.3390/diagnostics13203276)
Supplement: Supplementary file 1 [file diagnostics-13-03276-s001.zip › Supplementary table S1.pdf]

Supplementary Table S1. Comparisons of other laboratory results between the bronchoscopy group and non-bronchoscopy group.

**Blood biochemical parameters**			
**Glu (mmol/L)**			
Pre treatment	10.3(7.68,13.8)	9.2(6.9,14.8)	0.577
Post treatment	5.8(4.9,7.3)	8.7(5.86,11.5)	**<0.001**
P value	**<0.001**	**0.070**	
**ALT (u/L)**			
Pre treatment	47.4(24.575,108.175)	53.3(38.9,60)	0.445
Post treatment	32.8(16.3,61.8)	33.6(21.55,62.05)	0.476
P value	**<0.001**	**0.028**	
**AST (u/L)**			
Pre treatment	57.2(34.6,113.1)	60.4(48.55,76.85)	0.498
Post treatment	31.9(20.8,47.7)	37.2(23.85,70.1)	0.116
P value	**<0.001**	**0.049**	
**Urea (mmol/L)**			
Pre treatment	12.1(8,20.3)	15.3(9.5,20.36)	0.123
Post treatment	8.5(4.8,15.1)	11.4(6.735,21.68)	**0.037**
P value	**<0.001**	0.272	
**Cr (ummol/L)**			
Pre treatment	90.8(51.3,139)	113(64.6,184.4)	0.077
Post treatment	61(41,95)	115(65.45,230.55)	**<0.001**
P value	**<0.001**	0.955	
**Alb (g/L)**			
Pre treatment	31.8(28.8,35)	33.7(28.95,36.2)	0.126
Post treatment	34.9798±6.83641	30(25.75,35.1)	**<0.001**
P value	**<0.001**	**0.011**	
**myocardium markers**			
**TnI (ng/mL)**			
Pre treatment	0.09(0.016,0.7950)	0.046(0.0116,0.2155)	0.143
Post treatment	0.014(0.004,0.198)	0.127(0.034,0.3575)	**<0.001**
P value	**<0.001**	**0.049**	
**BNP (pg/ml)**			
Pre treatment	232(90.5,598.5)	96.45(46.635,643.7)	0.131
Post treatment	105(31.3,286.7)	455.72(74.38,1331.05	**<0.001**
P value	**<0.001**	**0.002**	
**D-dimer (mg/L)**			
Pre treatment	4.18(1.68,11.12)	5.56(4.005,6.700)	0.154
Post treatment	1.36(0.86,2.40)	2.11(1.325,6.545)	**<0.001**
P value	**<0.001**	**0.002**	

Abbreviations: ALT: alanine aminotransferase; AST: asperate aminotransferase; Cr:

creatinine; Alb: albumin; TnI: troponin I; BNP: brain natriuretic peptide.
