# Supplementary material for: Brochoscopic Airway Clearance Therapy vs. Conventional Sputum Aspiration: The Future of Flexible Brochoscopes in Intensive Care Units?
Source: Diagnostics (Basel). 2023 Oct 22;13(20):3276. doi: 10.3390/diagnostics13203276 (PMC10606468; doi:10.3390/diagnostics13203276)
Supplement: Supplementary file 1 [file diagnostics-13-03276-s001.zip › Supplementary table S2.pdf]

Supplementary Table S2. Median survival days and 14,28,90-day of survival rates in hospital between different groups.

	**Median survival days**	**14-day of survival rate (%)**	**28-day of survival rate (%)**	**90-day of survival rate (%)**
**Have bronchoscopy**	38	91.7	71.9	17.1
**No bronchoscopy**	17	60.5	24.3	4.9
**≤once every 3 days**	53	94	91.8	26.6
**>once every 3 days**	26	89.1	46.3	3.9
