# Supplementary figures and images for: Brochoscopic Airway Clearance Therapy vs. Conventional Sputum Aspiration: The Future of Flexible Brochoscopes in Intensive Care Units?
Source: Diagnostics (Basel). 2023 Oct 22;13(20):3276. doi: 10.3390/diagnostics13203276 (PMC10606468; doi:10.3390/diagnostics13203276)

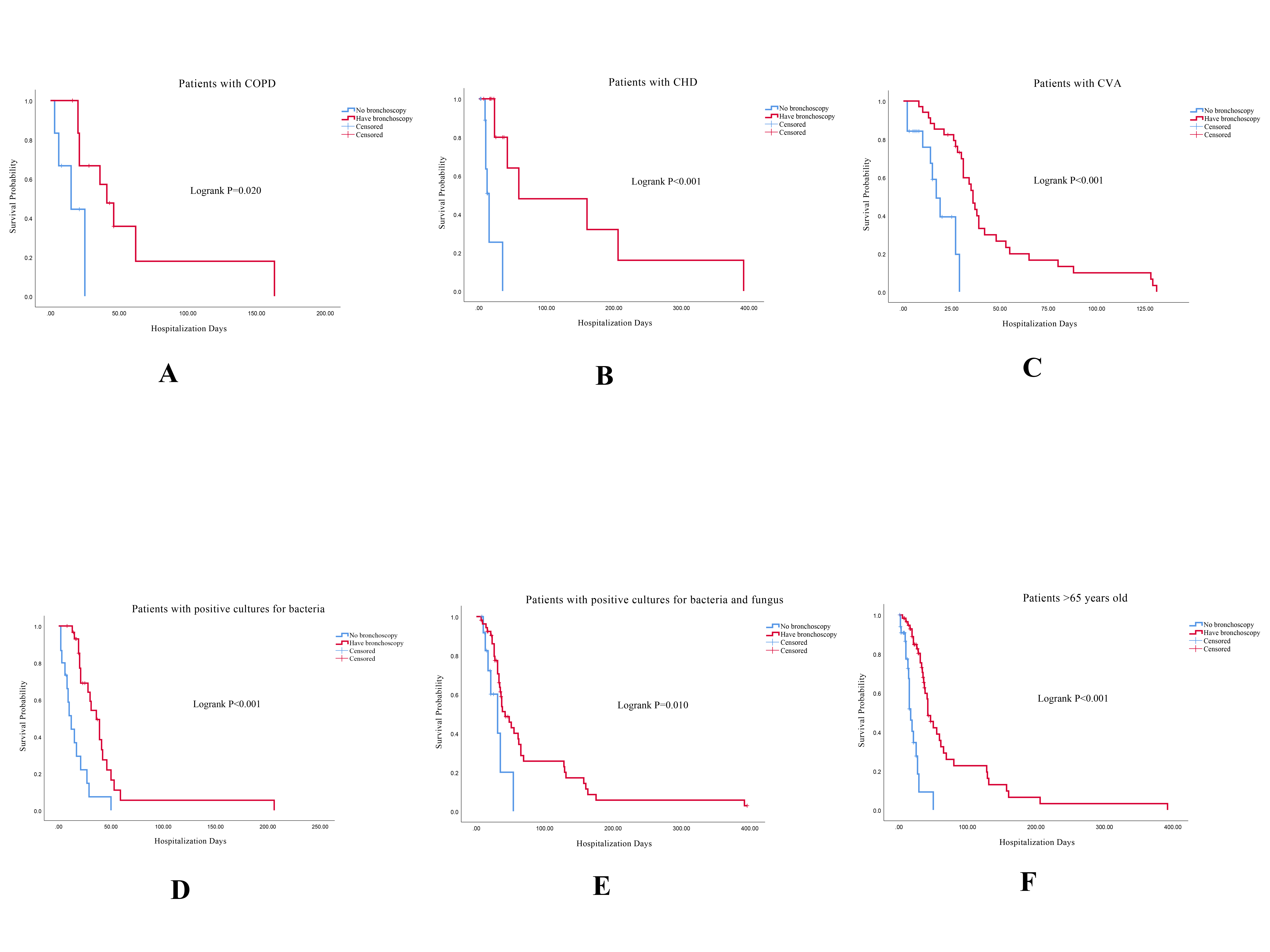

Supplement: Supplementary file 1 [file diagnostics-13-03276-s001.zip › Supplementary figure S1.tif]

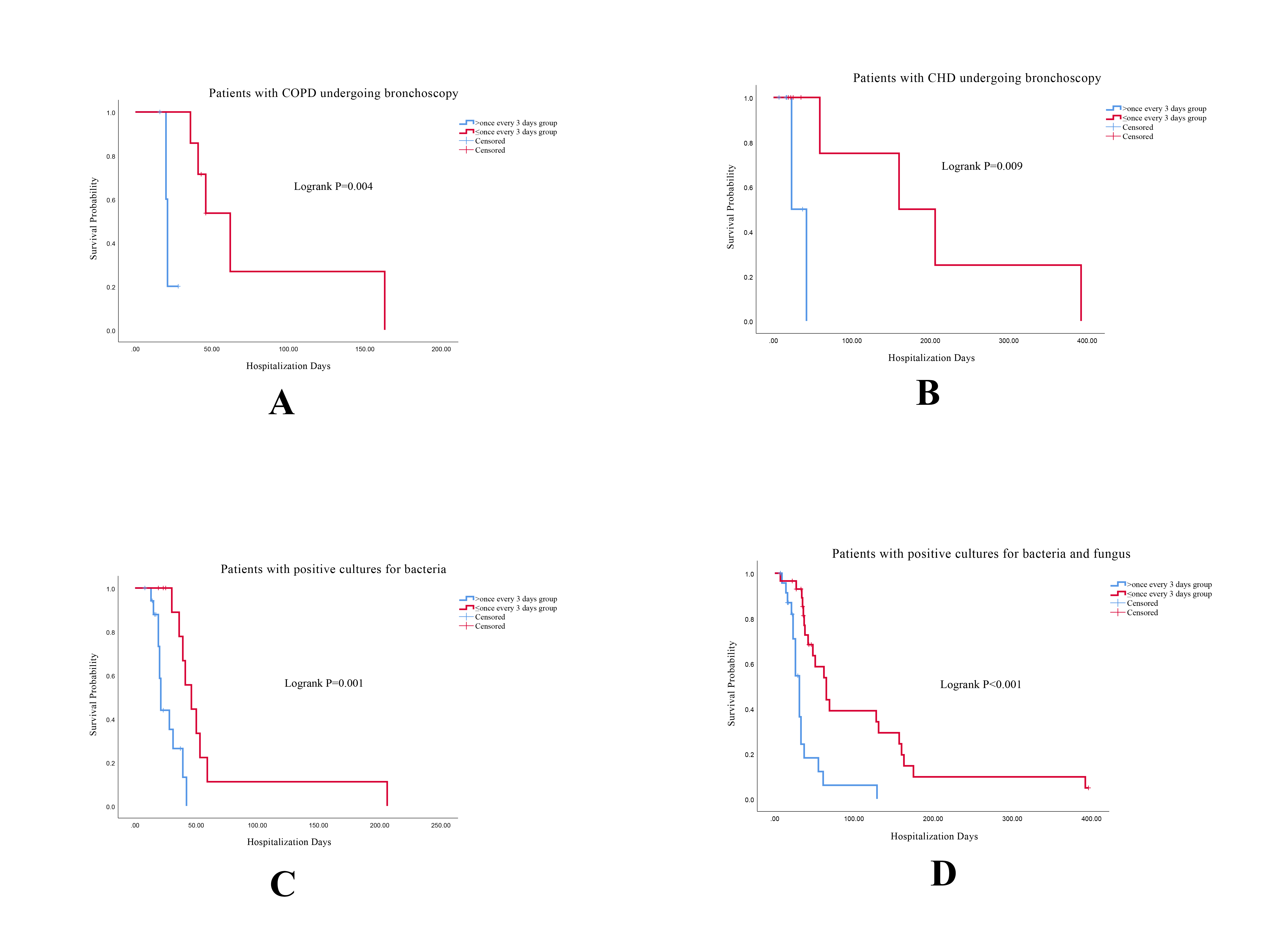

Supplement: Supplementary file 1 [file diagnostics-13-03276-s001.zip › Supplementary figure S2.tif]
